# *Bacillus thuringiensis* Suppresses Bacterial wilt Disease Caused by *Ralstonia solanacearum* with Systemic Induction of Defense-Related Gene Expression in Tomato

**DOI:** 10.1264/jsme2.ME12162

**Published:** 2012-12-19

**Authors:** Mitsuro Hyakumachi, Mitsuyoshi Nishimura, Tatsuyuki Arakawa, Shinichiro Asano, Shigenobu Yoshida, Seiya Tsushima, Hideki Takahashi

**Affiliations:** 1 Faculty of Applied Biological Sciences, Gifu University, Gifu 501–1193, Japan; 2 Graduate School of Agriculture, Hokkaido University, Sapporo 060–8589, Japan; 3 Environmental Biofunction Division, National Institute for Agro-Environmental Sciences, Tsukuba, Ibaraki 305–8604, Japan; 4 Graduate School of Agricultural Science, Tohoku University, Sendai 981–8555, Japan

**Keywords:** *Bacillus thuringiensis*, induced resistance, *Ralstonia solanacearum*, tomato

## Abstract

*Bacillus thuringiensis* is a naturally abundant Gram-positive bacterium and a well-known, effective bio-insecticide. Recently, *B. thuringiensis* has attracted considerable attention as a potential biological control agent for the suppression of plant diseases. In this study, the bacterial wilt disease-suppressing activity of *B. thuringiensis* was examined in tomato plants. Treatment of tomato roots with *B. thuringiensis* culture followed by challenge inoculation with *Ralstonia solanacearum* suppressed the development of wilt symptoms to less than one third of the control. This disease suppression in tomato plants was reproduced by pretreating their roots with a cell-free filtrate (CF) that had been fractionated from *B. thuringiensis* culture by centrifugation and filtration. In tomato plants challenge-inoculated with *R. solanacearum* after pretreatment with CF, the growth of *R. solanacearum* in stem tissues clearly decreased, and expression of defense-related genes such as PR-1, acidic chitinase, and *β*-1,3-glucanase was induced in stem and leaf tissues. Furthermore, the stem tissues of tomato plants with their roots were pretreated with CF exhibited resistance against direct inoculation with *R. solanacearum*. Taken together, these results suggest that treatment of tomato roots with the CF of *B. thuringiensis* systemically suppresses bacterial wilt through systemic activation of the plant defense system.

Pretreatment of plants with non-pathogenic micro-organisms, including symbiotic and endophytic fungi and bacteria, can induce resistance to pathogen infection (22). Plant growth-promoting rhizobacteria (PGPR) that colonize plant roots and exert a beneficial effect on plant growth are well known for their potential to reduce plant pathogen populations in the soil, thereby suppressing diseases (13, 44, 48). Some endophytes and arbuscular mycorrhizal fungi also protect plants or increase tolerance to pathogen infection (2, 33, 46). Furthermore, such microorganisms have been commercially used as biological control agents (BCAs) against pathogens (6). Disease suppression by BCAs seems to be caused by their direct or indirect interactions with pathogens, including mycoparasitism, production of antimicrobial substances, and competition for nutrients and space. In addition to these antagonistic effects on plant pathogens, evidence is emerging that BCAs produce elicitors that activate plant defense reactions (47). The reduction of diseases through this plant-mediated resistance mechanism is referred to as induced systemic resistance.

*Bacillus* spp. have been focused on as potential BCAs. So far, specific strains of the species *Bacillus amyloliquefaciens*, *Bacillus subtilis*, *Bacillus pasteurii*, *Bacillus. cereus*, *Bacillus pumilus*, and *Bacillus mycoides* have been reported to elicit significant reductions in the incidence or severity of various diseases in a diversity of hosts. Several *Bacillus* spp. are known to produce antibiotic cyclic lipopeptides that can suppress one or more pathogens (37). In addition, *B. cereus* strain UW85 produces two fungistatic antibiotics, zwittermicin A and kanosamine, which are effective in protecting alfalfa seedlings from damping-off caused by *Phytophthora medicaginis* (9, 35, 36). Many *Bacillus* spp. can also produce certain types of degrading enzymes. For example, *Bacillus ehimensis* produces chitin-degrading enzymes (11), and *B. subtilis* strain AF1 displays some fungitoxicity through the secretion of *N*-acetyl gluco-saminidase and glucanase (16). On the other hand, experimental proof concerning nutrient competition by *Bacillus* spp. is rare, although suppression of soil-borne plant pathogens through competition for nutrients has been demonstrated in some instances for some beneficial bacteria such as *Pseudomonas* (8).

Some *Bacillus* spp. can apparently activate the plant defense system, thereby suppressing the diseases caused by various pathogens. For example, treatment of sugar beet with *B. mycoides* induced the activity of new isoforms of *β*-1,3-glucanase and peroxidase, which are typical defense markers for induced resistance, and significantly reduced the severity of *Cercospora* leaf spot (1). The severity of blue mold of tobacco, caused by *Peronospora tabacina*, was reduced by treatment with *B. pasteurii* or *B. pumilus*, which can elicit induced resistance (51, 52, 53). Furthermore, in tomato plants treated with a combination of *B. subtilis* with some other strains of *Bacillus* spp., such as *B. pumilus* and *B. amyloliquefaciens*, the severity of viral disease caused by *cucumber mosaic* virus (CMV) was reduced because of induced resistance (12, 17).

*Bacillus thuringiensis* has been used as an effective bio-insecticide because it produces the proteins Cry and Cyt, which are highly toxic to insects, but not to mammals, and are not harmful to the environment (28, 34). Recently, *B. thuringiensis* has also attracted considerable attention as a biological control agent to suppress plant diseases (54). Disease suppression by *B. thuringiensis* is thought to be caused by antimicrobial substances produced in response to plant pathogens (3–5, 26, 27). Indeed, *B. thuringiensis* can produce extracellular compounds such as b-exotoxins and the antibiotic zwittermicin A (54); however, to our knowledge, the potential for disease suppression via resistance induced by *B. thuringiensis* has not been reported. In this study, the potential activity of *B. thuringiensis* for suppressing bacterial wilt in tomato through the induction of plant defense system was examined.

## Materials and Methods

### Growth conditions of plants and bacteria

*Solanum lycopersicum* cv. ‘Oogata-fukuju’ was grown in a cell-tray filled with cultured soil mix (Kureha, Tokyo, Japan) at 28°C in a growth chamber under 14 h light (70 μmol m^−2^s^−1^):10 h dark conditions. After three weeks, the plants were transferred with the soil mix into pots 9 cm in diameter and grown at 28°C in a greenhouse under natural light conditions. To analyze the defense gene expression, *S. lycopersicum* cv. ‘Oogata-fukuju’ was grown in quartz sand at 28°C in a growth chamber under continuous fluorescent light (70 μmol m^−2^s^−1^) and fertilized with 1,000-fold-diluted Hyponex solution (Hyponex Japan, Osaka, Japan) at 3-day intervals.

*Bacillus thuringiensis* serovars *fukuokaensis* B88-82, *sotto* RG1-6, *indiana* RG5-17, *israelensis*, *japonensis* N141 and *tohokuensis* were cultured in Nutrient Broth (NB) medium (Nissui, Tokyo, Japan) without NaCl at 28°C for 2 days. *Ralstonia solanacearum* isolate SUPP100 (race 1, biovar 4) belonging to phylotype I (15) was used for challenge inoculations. *R. solanacearum* was cultured at 30°C on PSA medium containing 5 g L^−1^ of Ca(NO_3_)·4H_2_O, 2 g L^−1^ Na_2_HPO_4_·12H_2_O, and 5 g L^−1^ peptone for 48 h.

### Treatment with *B. thuringiensis*

The culture of *B. thuringiensis* was adjusted to a final density of 1.8 × 10^8^ cfu mL^−1^ with sterilized distilled water. Aliquots of this bacterial culture (BC) were applied to plant roots as indicated below. Using the remainder of the BC, bacterial cells were briefly removed by centrifugation at 7,000 rpm for 10 min at 25°C. The pellet of bacterial cells was resuspended in distilled water (DW) and adjusted to a final density of 1.8 × 10^8^ cfu mL^−1^ and subsequently referred to as “bacterial cell suspension” (BS). The supernatant was filtered through a nitrocellulose membrane (0.22 μm pore size) and referred to as “filter-sterilized cell-free filtrate” (CF). The BS and CF were also applied to plant roots, respectively, as indicated below.

When the three-week-old plants were transferred to 9 cm pots, 30 mL each of the BC, BS, or CF solution of *B. thuringiensis* was poured into the pots. As a control, three-week-old plants were treated with DW. Five days after transplantation, the plants were challenge-inoculated with 1 × 10^7^ cfu mL^−1^ *R. solanacearum* as described below.

For RNA extraction, three-week-old plants were grown in quartz sand and carefully removed so as to minimize injury to the root tissues. After rinsing the roots with DW three times, they were dipped in 50 ml of the *B. thuringiensis* BC, BS, or CF for 48 h at 25°C. As a control, three-week-old plants were treated with DW for 48 h at 25°C

### Inoculation with *Ralstonia solanacearum* and disease assessment

*Ralstonia solanacearum* isolate SUPP100 was used for challenge inoculations. The bacterial cells were collected by centrifugation at 7,000 rpm for 10 min at 25°C and resuspended in DW to a final density of 1 × 10^7^ cfu mL^−1^. Twenty milliliters of the bacterial suspension was poured into each pot in which *B. thuringiensis*-treated plants were grown. The inoculated plants were grown at 30°C in the greenhouse for either 14 or 40 days. Disease severity, based on foliar symptoms of wilting, was monitored daily for 14 days after inoculation with the pathogen. Disease severity was assessed using a scale of 0–5: 0, healthy; 1, partial wilting of one lower leaf; 2, wilting of two to three lower leaves; 3, wilting of all but the top two to three leaves; 4, wilting of all leaves; or 5, dead. Disease severity was calculated using the formula:

(5A+4B+3C+2D+E)/5N×100

in which A=number of plants on scale 5; B=number of plants on scale 4; C=number of plants on scale 3; D=number of plants on scale 2; E=number of plants on scale 1; N=total number of plants. Each experiment consisted of three replicates per treatment, and four plants per replicate were inoculated with pathogen. Plants were arranged in the growth chamber in a completely randomized design. All data from the repeated trials were pooled because variances were homogeneous. Data were subjected to analysis of variance and treatment means were compared by either Fisher’s least significant difference test or Student’s *t*-test.

Forty days after the challenge inoculation with *R. solanacearum*, the appearance of necrotic symptoms was carefully observed on stem sections. To analyze bacterial populations in stem tissues, two stem fragments, one 30 mm in length from near the root base and another 20 mm in length and ~30–50 mm above the root base, were excised from four plants after the challenge inoculation and ground with DW in a mortar and pestle. The original solution and 10-fold serial dilutions of the homogenate were spread onto three plates of Hara-Ono medium (10). The colonies were counted after 2 days of incubation at 30°C.

To directly analyze the resistance to *R. solanacearum* in the stem tissues, the stems of plants whose roots had been pretreated with the CF of *B. thuringiensis* or DW as a control were challenge-inoculated using a needle soaked in 1 × 10^7^ cfu mL^−1^ *R. solanacearum* isolate SUPP100, just above the cotyledons.

### Defense gene expression analysis

Total RNA was extracted from leaf, stem, and root tissues of tomato plants using the RNeasy Plant Mini kit (Qiagen, Hilden, Germany) according to the manufacturer’s instructions. First-strand cDNA was synthesized using PrimeScript RT reagent with the gDNA Eraser kit (Takara-Bio, Otsu, Japan). For northern hybridization analysis, total RNA (15 μg) was loaded into each lane on a 1.2% denaturing agarose gel. Northern hybridization analysis was performed according to Sambrook and Russell (32). To detect the expression of *PR-1*(*P6*), ~1000 bp fragments of the gene were amplified by PCR with the primers 5′-CATAACGATGCCCCGT GCCCAAGTCGG-3′ and 5′-GTAAGGACGTTGTCCGATCCA GTTGCC-3′ for *PR-1*(*P6*) (42). One microgram of first-strand cDNA was added to 50 μL of 10 mM Tris-HCl (pH 8.3) containing 50 mM KCl; 2 mM MgCl_2_; 0.2 mM each of dATP, dCTP, dGTP, and dTTP; 0.2 μm of each primer; and 5 units of KOD-plus DNA polymerase (Toyobo, Osaka, Japan) for PCR. The reaction was run with the following program: 30 cycles at 95°C for 30 s, 55°C for 1 min, and 72°C for 2 min. The PCR product was purified and cloned into the *Eco*RV site of pBluescript SK+ (Stratagene, La Jolla, CA, USA) according to the procedure of Takahashi and Ehara (38). To confirm that the expected DNA was cloned, the nucleotide sequence of each insert was determined by the Sanger method using a CEQ 8000 automated DNA sequencer (Beckman Coulter, Fullerton, CA, USA). The PCR probe for *PR-1*(*P6*) was labeled with digoxigenin (DIG)-11-dUTP using a DIG PCR labeling kit (Roche, Mannheim, Germany). DIG-labeled probe was detected using an alkaline phosphatase-conjugated anti-DIG antibody (Roche) and was visualized with the chemiluminescent substrate CDP-Star according to the manufacturer’s instructions (New England Biolabs, Beverly, MA, USA).

Expression of acidic chitinase (*Acht*), *β*-1,3-glucanase (*Bgl*), and actin (*Act*) genes in the leaves of tomato plants was analyzed by semi-quantitative RT-PCR 48 h after roots were treated with either the CF from *B. thuringiensis*, benzo(1,2,3)thiadiazole-7-carbothioic acid S-methyl ester (BTH) as a positive control, or DW as a negative control. Semi-quantitative RT-PCR amplification was performed in a 20 μL reaction volume containing 1 μL of cDNA diluted 10-fold with 1×KOD buffer provided by the manufacturer (Toyobo); 0.2 μM of each primer; 0.2 mM each of dATP, dGTP, dCTP, and dTTP; 1 mM MgSO_4_; and 1 unit KOD-plus-DNA polymerase (Toyobo), and run with the following program: 55°C for 2 min, followed by 20–30 cycles at 95°C for 30 s, 55°C for 30 s, and 68°C for 2 min. For RT-PCR of *Achi* and *Bgl* transcripts, the following set of primers was used: Achi-F (GCACTGTCTTGTCTCTTTTTC) and Achi-R (ATGGTTTATTATCCTGTTCTG) for *Achi*; and Bgl-F (ATTGT TGGGTTTTTGAGGGAT) and Bgl-R (TTTAGGTTGTATTTTG GCTGC) for *Bgl*. As an internal standard control, the level of *Act* transcript was amplified by RT-PCR using the following set of set of primers: Act-F (GGGGAGGTAGTGACAATAAATAACAA) and Act-R (GACTGTGAAACTG-CGAATGGC). Five microliter samples of PCR products were separated by gel electrophoresis on 1.5% agarose, stained with ethidium bromide, and visualized under UV light according to the standard protocol.

## Results

### Suppression of bacterial wilt in tomato treated with a bacterial culture (BC) of *B. thuringiensis*

To investigate the potential activity of *B. thuringiensis* for suppressing bacterial wilt in tomato, caused by *R. solanacearum*, the roots of tomato plants were treated with bacterial culture (BC) of *B. thuringiensis* serovars *fukuokaensis* B88-82, *sotto* RG1-6, *indiana* RG5-17, *israelensis*, *japonensis* N141, *tohokuensis* or a distilled water (DW) control, then challenge-inoculated with *R. solanacearum*. Among those six serovars, *fukuokaensis* B88-82 and *sotto* RG1-6 had stable activity of disease suppression (data not shown). Thus, we focused on those two serovars to further analyze the mechanism of the suppression of bacterial wild disease.

**Fig. 1:**
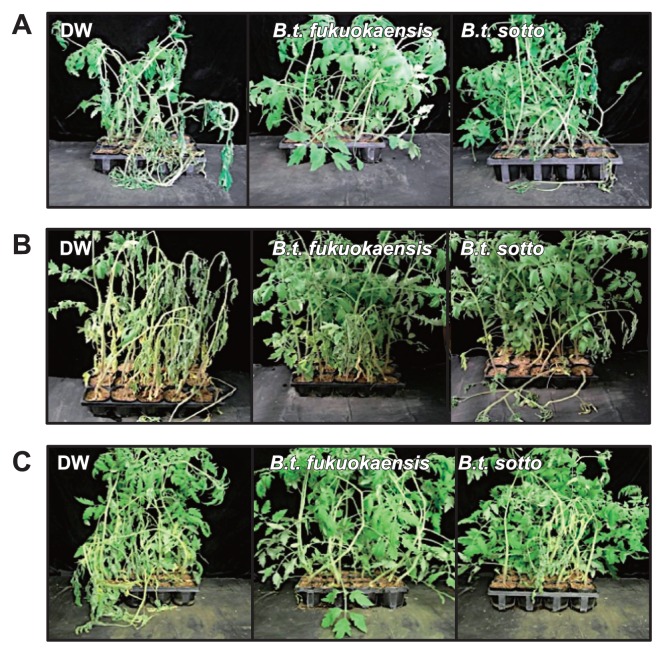
Development of bacterial wilt symptoms in tomato plants with their roots treated with *Bacillus thuringiensis*. The roots of three-week-old plants were soaked in 50 mL bacterial culture (BC) of *B. thuringiensis* serovars *fukuokaensis* B88-82 and *sotto* RG1-6 (A), bacterial cell suspension (BS) (B) or filter-sterilized cell-free filtrate (CF) (C). BC and CF were prepared by collecting the pellet of bacterial cells and the supernatant by centrifugation, respectively. As a control, three-week-old plants were treated with DW.

**Fig. 2:**
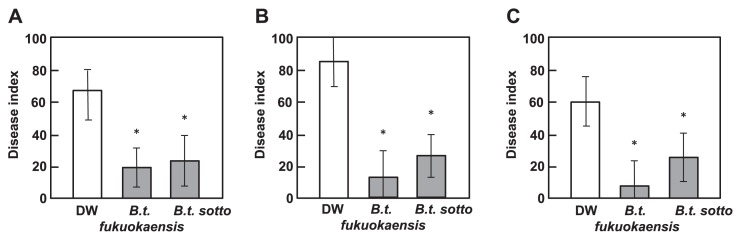
Severity of bacterial wilt diseases in tomato plants with their roots treated with *B. thuringiensis*. The roots of three-week-old plants were treated with the bacterial culture (BC) of *B. thuringiensis* serovars *fukuokaensis* and *sotto* (A), bacterial cell suspension (BS) (B) or filter-sterilized cell-free filtrate (CF) (C). As a control, three-week-old plants were treated with DW. Each experiment consisted of three replicates per treatment and four plants per replicate were inoculated with pathogen. Asterisk (*) indicates a statistically significant difference in disease severity between DW-treated and BC-, BS-, or CF-treated plants.

**Fig. 3:**
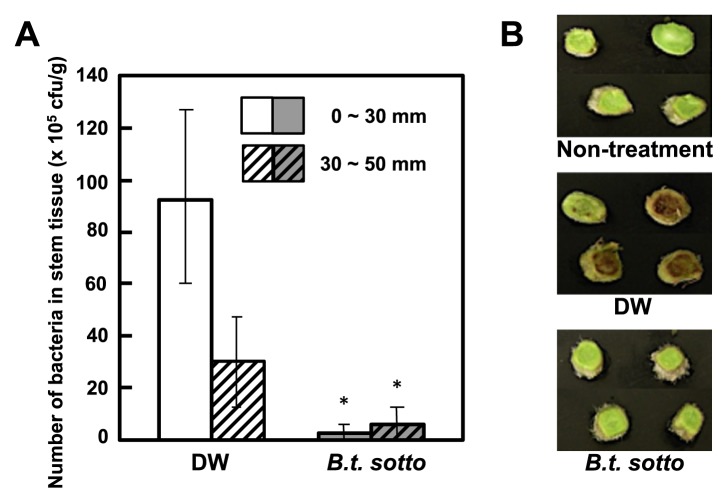
Necrotic symptom appearance and bacterial growth in the stem tissues of tomato plants with their roots treated with the bacterial culture (BC) of *B. thuringiensis*. The roots of three-week-old plants were treated with the bacterial culture (BC) of *B. thuringiensis* serovar *sotto*. As a control, three-week-old plants were treated with DW or were not treated at all (non-treated). Five days after transplantation, the plants were inoculated with *Ralstonia solanacearum*. (A) Forty days after inoculation, bacterial population numbers in 30 mm stem sections from near the root base (column in white or gray), or 20 mm in length from sections 30–50 mm above the root base (column with white or gray stripe) were measured. Each experiment consisted of three replicates per treatment and four plants per replicate were inoculated with pathogen. Asterisk (*) indicates a statistically significant difference in disease severity between DW-treated and BC-treated plants. (B) Four stem sections 50 mm from the root base of BC- or DW-treated tomato plants or non-treated control were photographed.

Fourteen days after inoculation with *R. solanacearum*, the development of wilt symptoms was observed in DW-treated control plants, but not in plants treated with either BC of *B. thuringiensis* serovar *fukuokaensis* B88-82 or *sotto* RG1-6 (Fig. 1A and 2A). In BC-treated plants, the growth of *R. solanacearum* in the stem tissues, moving from the initially inoculated root tissues, was significantly suppressed in comparison with those in DW-treated plants (Fig. 3A). Furthermore, the appearance of necrosis in sections of the stems, which seemed to be caused by the growth of *R. solanacearum*, was hardly observed in tomato plants whose roots were pretreated with the *B. thuringiensis* BC, whereas necrosis clearly developed on sections of stems with bacterial growth in the DW-treated control (Fig. 3A, B).

To characterize the disease-suppressive activity of *B. thuringiensis*, the BC of *B. thuringiensis* was fractionated into a bacterial cell suspension (BS) and a filter-sterilized cell-free filtrate (CF) by centrifugation and filtration. When tomato roots were treated with the BS or CF of *B. thuringiensis*, followed by challenge inoculation with *R. solanacearum*, the development of wilt symptoms was significantly suppressed by treatment not only with the BS but also with the CF of *B. thuringiensis* (Fig. 1B, C and Fig. 2B, C).

### Induction of defense-related gene expression in tomato treated with *B. thuringiensis*

**Fig. 4:**
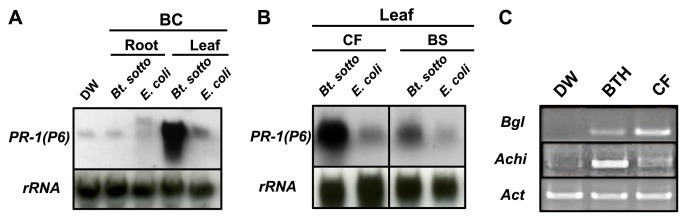
Expression of defense-related genes in tomato plants with their roots treated with *B. thuringiensis*. (A) Transcripts of tomato *PR-1*(*P6*) in the root and leaf tissues of tomato plants with their roots treated with the bacterial culture (BC) of *B. thuringiensis*, or *E. coli* or DW as controls, were detected by northern hybridization. rRNA was used as an internal control of loading RNA sample. (B) Transcripts of tomato *PR-1*(*P6*) in the leaf tissues of tomato plants with their roots treated with the bacterial suspension (BS) or cell-free filtrate (CF) of *B. thuringiensis*, or *E. coli* as a control, were detected by northern hybridization. (C) Induction of acidic chitinase (*Acht*) and β-1,3-glucanase (*Bgl*) genes in the leaves of tomato plants at 48 h after treatment of their roots with the CF of *B. thuringiensis*, benzo(1, 2, 3)thiadiazole-7-carbothioic acid S-methyl ester (BTH) as a positive control, or DW as a negative control, was analyzed by semi-quantitative RT-PCR. Actin (*Act*) transcripts were also amplified as an internal standard control.

To explore whether the CF of *B. thuringiensis* can activate induced resistance in plants, the expression of defense-related genes in tomato plants treated with *B. thuringiensis* was investigated. When the expression of defense-related genes in tomato plants was analyzed by northern hybridization, the expression of tomato *PR-1*(*P6*) in leaf tissue was clearly induced by treatment of the roots with the BC of *B. thuringiensis* (Fig. 4A), whereas it was slightly induced by treatment with *E. coli* as a control. *PR-1*(*P6*) expression in leaf tissues was also induced in tomato plants whose roots were treated with BS and CF, but not in the *E. coli*-treated control (Fig. 4B). Furthermore, the up-regulation of *β*-1,3-glucanase (*Bgl*) and acidic chitinase (*Achi*) gene expression in the leaves of tomato plants whose roots were treated with the CF or in BTH-treated leaves was also confirmed by semi-quantitative RT-PCR (Fig. 4C).

### Suppression of bacterial wilt in *B. thuringiensis*-treated tomato plants inoculated with *R. solanacearum*

**Fig. 5:**
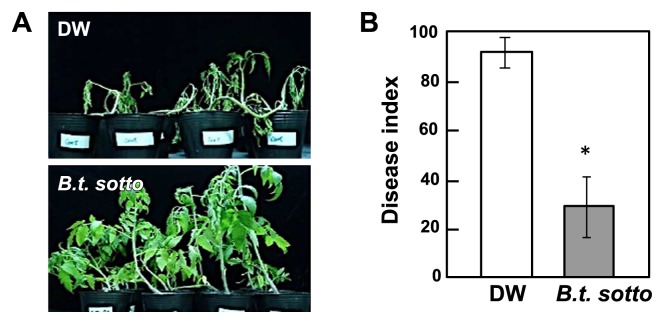
Development of bacterial wilt symptoms and disease severity in tomato plants with their roots treated with the cell-free filtrate (CF) of *B. thuringiensis* following needle inoculation of the stem tissues with *R. solanacearum*. (A) Development of bacterial wilt symptoms in tomato plants with their roots treated with the CF of *B. thuringiensis* serovar *sotto*. As a control, three-week-old plants were treated with DW. Five days after transplantation, the stems of the plants were needle-inoculated with *Ralstonia solanacearum* just above the cotyledons. (B) Severity of bacterial wilt disease in tomato plants with their roots treated with CF. Disease severity based on foliar symptoms of wilting was monitored daily for 14 days after inoculation with the pathogen. Each experiment consisted of three replicates per treatment, and four plants per replicate were inoculated with the pathogen. Asterisk (*) indicates a statistically significant difference in disease severity between DW-treated and CF-treated plants.

If systemic induction of defense-related gene expression by treatment with *B. thuringiensis* CF is associated with suppression of bacterial wilt caused by *R. solanacearum*, the above-ground part of tomato plants should exhibit enhanced resistance to *R. solanacearum*. To examine enhanced resistance to *R. solanacearum* in the stem tissues of tomato plants with their roots treated with the CF of *B. thuringiensis*, the stems of CF-treated plants were directly needle-inoculated with *R. solanacearum*. The development of wilt symptoms was not observed in CF-treated plants but was observed in control plants (Fig. 5A). Furthermore, the severity of wilt disease was significantly suppressed in CF-treated plants (Fig. 5B), indicating that the stem tissues exhibit enhanced resistance to *R. solanacearum*.

### Differential induction of *PR-1* expression in leaf, stem, and root tissues of tomato plants treated with *B. thuringiensis*

**Fig. 6:**
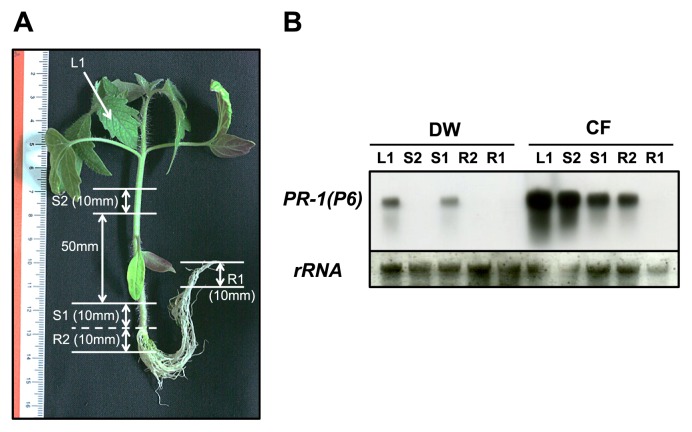
Differential expression of the *pathogenesis-related 1* [*PR-1*(*P6*)] gene in leaf, stem, and root tissues of tomato plants with their roots treated with the cell-free filtrate (CF) of *B. thuringiensis*. (A) Tomato plant used for RNA extraction. For northern hybridization, total RNA was isolated from leaf tissue (L1), two stem sections 10 mm in length from near the root base (S1), a stem section 10 mm in length located 50–60 mm above the root base (S2), two root sections 10 mm in length from under the soil surface (R2), and a 10 mm section including root tip (R1). (B) Transcripts of tomato *PR-1*(*P6*) in the leaf (L1), stem, (S1 and S2) and root (R1 and R2) tissues of tomato plants treated on their roots with the CF of *B. thuringiensis* were detected by northern hybridization. A DW-treated tomato plant was used as a control. rRNA was used as an internal control for loading RNA samples.

As the expression of defense-related genes was up-regulated in the leaves of tomato plants with CF-treated roots, we further analyzed systemic induction of defense gene expression in tomato plants (Fig. 6). For northern hybridization, total RNA was isolated from plant tissues, including leaf, stem, main root, or lateral root, shown in Fig. 6A, after dipping the main and lateral roots into CF for 48 h. *PR-1*(*P6*) expression was clearly induced in leaf, stem, and main root tissues, but not in lateral roots (Fig. 6B), although both main and lateral root tissues were dipped in CF.

## Discussion

*Bacillus thuringiensis* can suppress the growth of *R. solanacearum* and the development of wilt symptoms in tomato plants. Because bacterial wilt caused by *R. solanacearum* was also significantly suppressed by treating the roots with the CF of *B. thuringiensis*, the suppressive activity is likely to be not caused by competition between *B. thuringiensis* and *R. solanacearum* for nutrients or space in the soil. *Bacillus thuringiensis* generally produces several compounds, including antimicrobial substances that include b-exotoxins, antibiotics, degrading enzymes, bacteriocins, and a signal molecule in the bacterial quorum-sensing system (3–5, 17, 26, 54). Thus, the suppression of wilt disease in tomato treated with *B. thuringiensis* BS may be due to the activity of extracellular compounds produced by *B. thuringiensis* cells in soil; however, when *R. solanacearum* was cultured on PSA medium containing the CF, bacterial growth was not inhibited (unpublished result). Therefore, there is less possibility that such antimicrobial substances included in the CF may directly suppress the growth and spread of *R. solanacearum* in tomato. On the other hand, in not only CF-treated root tissues but also untreated stem tissues in which defense-related gene expression was clearly induced, the growth of *R. solanacearum* was significantly suppressed. Thus, extracellular compounds secreted by *B. thuringiensis* into the CF may inhibit the growth and spread of *R. solanacearum* in stem tissues through activation of the plant defense system, rather than either antibacterial activity or nutrient competition. It has been reported that some *Bacillus* spp. strains produce elicitors that activate the plant defense system (1, 12, 14, 17, 51–53), although evidence supporting the ability of *B. thuringiensis* to activate the plant defense system has not been presented so far. This is the first report showing that bio-insecticidial *B. thuringiensis* activates the plant defense system in response to recognition of elicitor molecules included in the CF.

*R. solanacearum* generally invades root tissues through wound sites and grows in intercellular spaces to establish systemic infection. The bacteria spread rapidly throughout the vascular system, thereby inducing alteration of water fluxes (31, 45). In resistant tomato cultivars, bacteria were localized in primary xylem tissues in infected root tissues, whereas in susceptible cultivars, bacteria were found in both primary and secondary xylem tissues, and often in intercellular spaces of necrotic cells in xylem and nearby pith tissues (18–20). In this study, the suppression of *R. solanacearum* growth in stem tissues by treating the roots with the CF from *B. thuringiensis* was accompanied by systemic induction of defense gene expression. Therefore, in tomato plants with their roots treated with CF, the growth and spread of *R. solanacearum* are likely to be effectively suppressed by activation of defense reactions in xylem tissues, thereby controlling bacterial wilt disease.

Salicylic acid (SA), jasmonic acid (JA), and ethylene (ET) are involved to different extents in defense responses against a broad range of pathogens (7, 24). In general, the SA-dependent signaling pathway interacts antagonistically with the JA/ET-dependent signaling pathways (7, 21, 39), although basal resistance to certain pathogens is controlled by the combined actions of SA, JA, and ET-dependent signaling pathways (49). Treatment of plant roots with plant growth-promoting rhizobacteria (PGPR) systemically enhances resistance to pathogens by activating the plant defense system or induced systemic resistance (ISR) (23, 25, 29, 30, 43, 50). ISR elicited by PGPR is mainly mediated by JA/ET-dependent signaling pathways (40, 41), although recently, evidence indicating partial involvement of the SA-dependent signaling pathway was presented in some cases. However, in tomato plants with *B. thuringiensis* CF-treated roots, the expression of SA-responsive defense-related genes was induced primarily (Fig. 4 and 6). Thus, treatment with *B. thuringiensis* may be differentially effective for induced resistance to pathogens compared with treatment with PGPR.

*Bacillus thuringiensis* is best known as an insecticide. The disease suppressive activity of *B. thuringiensis* against infection with plant pathogens indicates that *B. thuringiensis* can be used as a microbial biocontrol agent to suppress plant diseases. Thus, *B. thuringiensis* has potential as a bifunctional biopesticide to control a broad range of pests for plant protection. The disease-suppressive activity of *B. thuringiensis* and subsequent activation of the plant defense system presented in this study will contribute to further evaluation of the practicality of *B. thuringiensis* as an effective biocontrol agent.
